# Periostin within the tumor microenvironment: molecular insights and immunomodulatory roles in cancer

**DOI:** 10.3389/fimmu.2026.1869363

**Published:** 2026-10-30

**Authors:** Yongxin Zhao, Yan Yu, Shuangda Li, Lijuan Xu, Weizhi Chen, Jing Lv

**Affiliations:** Department of Clinical Laboratory, Honghui Hospital, Xi’an Jiaotong University, Xi’an, China

**Keywords:** biomarker, cancer−associated fibroblasts, immune checkpoint blockade, immunomodulation, periostin, therapeutic target, tumor microenvironment

## Abstract

Periostin, also known as osteoblast-specific factor 2 (OSF-2), is a secreted matricellular protein with pleiotropic functions in both physiological and pathological processes. While it contributes to tissue repair, bone remodeling, and cardiovascular and respiratory homeostasis, accumulating evidence highlights its critical role in cancer progression. In diverse malignancies—including esophageal, gastric, colorectal, pancreatic, hepatocellular, ovarian, lung, breast carcinomas, and glioma—periostin is frequently overexpressed in both the stromal and epithelial compartments. Its regulation is consistently correlated with increased tumor invasiveness, advanced clinical stage, poor prognosis, and shortened patient survival. Mechanistically, periostin promotes tumor progression through multiple interconnected pathways, including the activation of NF-κB and AKT signaling, induction of epithelial–mesenchymal transition, stimulation of angiogenesis, and maintenance of cancer stemness. Within the tumor microenvironment, periostin-expressing cancer-associated fibroblasts drive extracellular matrix remodeling, establish an immunosuppressive niche, and impede CD8^+^ T-cell infiltration, thereby attenuating antitumor immunity and limiting responses to immunotherapy. This review systematically summarizes the structure, expression, and multifaceted functions of periostin, with a particular focus on its immunomodulatory actions within the tumor microenvironment, and discusses its emerging potential as a prognostic biomarker and therapeutic target in cancer immunotherapy.

## 1 Introduction

Periostin (POSTN), also known as osteoblast-specific factor 2 (OSF-2), is an extracellular matrix protein composed of 836 amino acids. It was first identified in mouse MC3T3-E1 osteoblast-like cells through subtractive hybridization and differential screening (1). The human POSTN gene is located on chromosome 13q13.3, spans approximately 36 kb, contains 23 exons, and encodes a protein with a molecular weight of approximately 93 kDa (2). Under physiological conditions, periostin is expressed in various fetal and adult tissues—including the embryonic periosteum, placenta, and periodontal ligament—where it mediates cell-matrix interactions by binding through its fasciclin-1 (FAS1) domain (3, 4). Specifically, periostin binds to extracellular matrix components, such as fibronectin, tenascin-C, and collagen V, and periostin itself, thereby regulating cellular adhesion and signaling (3, 4). These interactions underpin the essential roles of periostin in embryonic morphogenesis, tissue repair, and the development and maintenance of bone, teeth, heart valves, and tendons (5).

In addition to its physiological functions, periostin is increasingly recognized as a key player in diverse pathologies. In orthopedic disorders, altered periostin expression is closely linked to disease states: reduced levels are associated with connective tissue defects, whereas elevated levels correlate with the progression and severity of osteoarthritis (6). Periostin also actively contributes to fracture healing and bone regeneration (7). In the cardiovascular and respiratory systems, periostin is markedly upregulated following acute myocardial infarction (AMI), where it promotes cardiomyocyte proliferation and inhibits apoptosis, thereby influencing postinfarct remodeling (8). Similarly, in pulmonary arterial hypertension (PAH), periostin is associated with disease progression through a hypoxia-induced positive feedback loop with HIF-1α and may serve as a biomarker for assessing disease severity (9).

Notably, periostin is frequently overexpressed in the tumor microenvironment—both in the stroma and, occasionally, in cancer epithelial cells. Its upregulation is closely associated with increased tumor invasiveness, advanced clinical stage, poor prognosis, and poor patient survival across multiple cancer types, highlighting its emerging dual role as a prognostic biomarker and a potential therapeutic target (10). This review systematically explores the structure, distribution, and functions of periostin, with a particular focus on its multifaceted roles in tumor biology and the tumor immune microenvironment.

To ensure a comprehensive and rigorous assessment of the current evidence, we systematically searched the PubMed, Web of Science, and Scopus databases for articles published between 2000 and early 2026. The search strategy combined keywords for “periostin” (or “POSTN”) with terms covering “cancer, “ “tumor microenvironment, “ “immunotherapy, “ “cancer-associated fibroblasts, “ “immune checkpoint, “ “prognosis, “ “metastasis, “ and “signaling pathway.” Priority was given to peer-reviewed original research, clinical studies, and high-quality reviews published in English, with emphasis on literature from the last decade while retaining seminal earlier works. Non-English articles, conference abstracts, and unpublished data were excluded. Where conflicting findings were identified, preference was given to studies with larger sample sizes or robust experimental designs (e.g., *in vivo* models or multivariate analyses). Additionally, contradictory or context-dependent observations were explicitly discussed to ensure a balanced perspective, rather than being harmonized into a single narrative.

## 2 Structure, tissue distribution and biological functions of periostin

### 2.1 Molecular structure

The amino acid sequence of periostin is approximately 90% homologous between mice and humans. The murine Postn gene is located on chromosome 3, whereas the human POSTN gene resides on chromosome 13; both genes contain 23 exons (11). A defining structural feature of periostin is the presence of four FAS1 domains (as illustrated in **Figure 1**), each consisting of two short, highly conserved amino acid motifs (12). The FAS1 domain is evolutionarily conserved across diverse species, including Drosophila fasciclin-1, human and mouse βig-h3, algal-CAM, and mycobacterial MPB70 (13). Through these domains, periostin interacts with various extracellular matrix (ECM) and cell-surface molecules, initiating downstream intracellular signaling cascades that regulate processes such as cell adhesion and migration (6).

**Figure 1:**
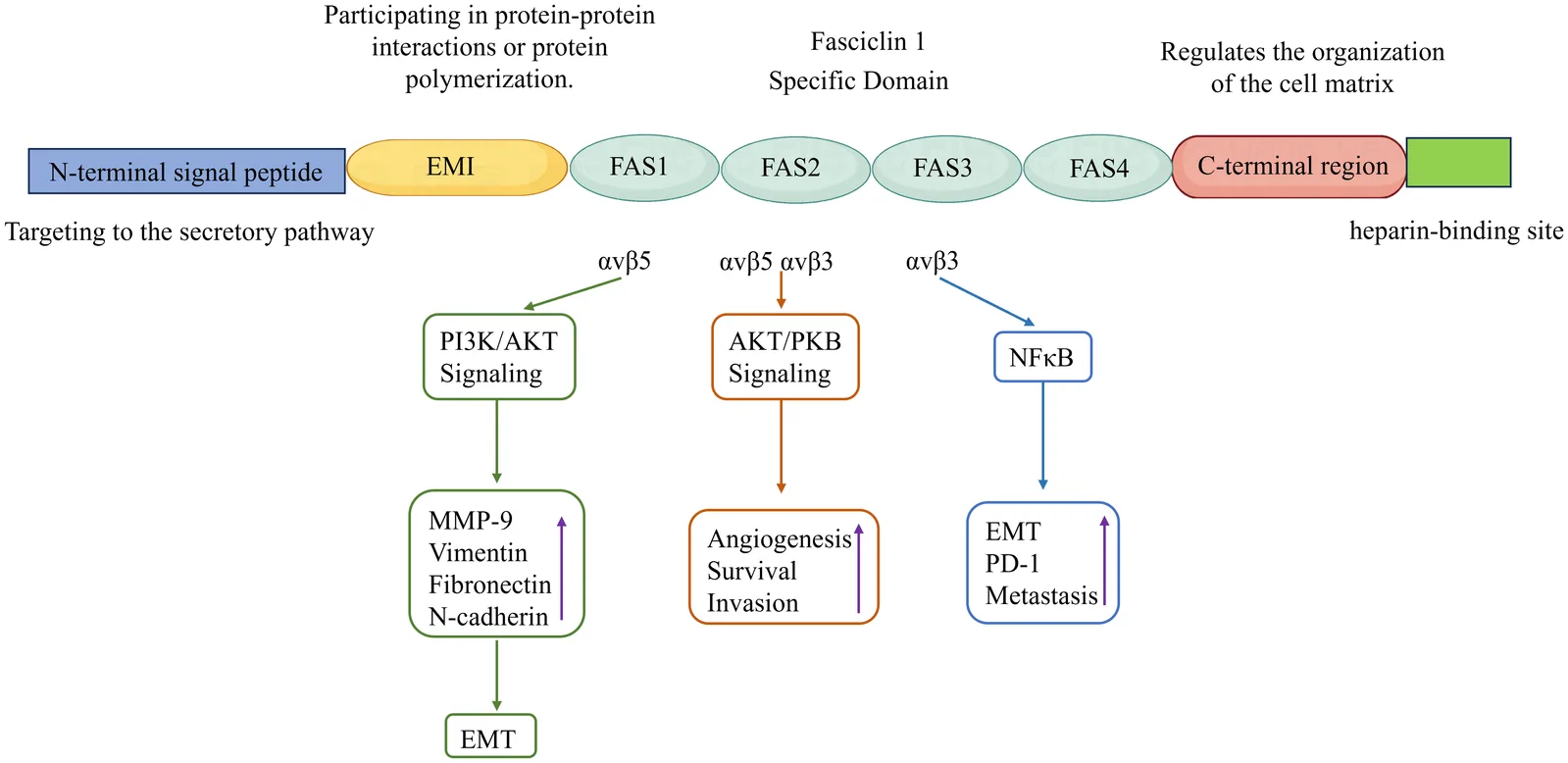
Schematic representation of periostin protein structure. Periostin comprises an N-terminal signal peptide, a cysteine-rich EMI domain, four tandem fasciclin-1 (FAS1) domains, and a C-terminal variable domain generated by alternative splicing. Heparin-binding sites (Hbs) are located within the C-terminal domain (CTD). Key interacting partners and downstream signaling pathways—including integrins, NF-κB, and PI3K/AKT—are indicated based on their binding regions.

Notably, compared with the more conserved N-terminal portion, the C-terminal region of periostin exhibits considerable variability in exon number, length, and splicing patterns across vertebrates (11). Alternative splicing in this region results in insertions or deletions, generating multiple splice variants that have been identified in humans (14). The N-terminal segment contains a signal peptide that is essential for protein secretion and extracellular localization, as well as an N-terminal cysteine-rich (EMI) domain (15). Encoded by exons 2 and 3, the EMI domain comprises approximately 75 amino acids and is thought to mediate protein-protein interactions or multimerization (5).

### 2.2 Tissue distribution

Initial immunohistochemical studies in mice revealed prominent periostin expression in vascularized, cell-rich connective tissues such as the periosteum and the periodontal ligament (13). Subsequent work has detected periostin in a wide range of normal human tissues, including heart valves, lungs, tendons, aorta, stomach, lower gastrointestinal tract, placenta, uterus, and breast (5).

### 2.3 Key physiological functions

In bone biology, periostin helps regulate bone mass and remodeling by modulating sclerostin levels; under physiological conditions, periostin suppresses sclerostin, thereby promoting bone formation and turnover (16). Conversely, periostin deficiency leads to elevated sclerostin levels, which disrupt normal remodeling and reduce bone mass (16). Different periostin isoforms are expressed at specific stages of embryogenesis and postnatal development, suggesting distinct roles in skeletal growth and maturation (17). In adults, periostin is re-expressed during fracture healing and in response to mechanical or metabolic stimuli, where it contributes to bone adaptation (18).

In addition to its role in the skeletal system, periostin plays essential roles during tooth development. It is detected early in epithelial and mesenchymal cells involved in mineralization and later localizes to the adult periodontal ligament and dental pulp at hard-soft tissue interfaces (19). Its absence in fully mineralized tissues implies a stage-specific regulatory role in odontogenesis (19). *In vitro*, periostin increases the migration, proliferation, and osteogenic differentiation of human periodontal ligament stem cells (20). As a key mediator of the biomechanical properties of fibrous connective tissue, periostin helps maintain periodontal architecture and ligament homeostasis (21).

Within the cardiovascular system, periostin is widely expressed in the fibrous cardiac skeleton and endocardial cushions during development but not in cardiomyocytes (22). It serves as a marker for mesenchymal cells after epithelial-mesenchymal transition in cushion tissue and persists in remodeled mature valves (22). During neonatal cardiac remodeling, peak periostin expression stimulates collagen deposition, tissue compaction, and fibroblast proliferation, thereby contributing to ventricular wall stiffening and valvular functional maturation (16). Periostin-expressing cells also support ECM maturation and are required for postnatal sympathetic nerve patterning in the heart (23). In vascular contexts, aortic injury upregulates periostin, which in turn can promote the calcification of vascular smooth muscle cells (24).

## 3 Implications of periostin in nonneoplastic diseases

In addition to its physiological roles, periostin is critically involved in the pathogenesis of several nonneoplastic disorders. In the musculoskeletal system, it is essential for development, fracture healing, and bone homeostasis, and has emerged as a potential prognostic biomarker and monitoring tool for orthopedic conditions such as osteoarthritis and rheumatoid arthritis (6). During skeletal muscle repair, periostin is expressed by fibroblasts and promotes the paracrine recruitment of distant fibroblasts, driving fibrotic scar formation and thereby impairing functional regeneration (25). Its expression is significantly elevated in both osteoarthritis and rheumatoid arthritis, and its levels are positively correlated with disease severity and radiographic progression (26–29).

Within the cardiovascular system, periostin is indispensable for cardiac development and postinjury remodeling, and contributes to cardiomyocyte adhesion, interstitial fibrosis, and valvular morphogenesis (30). Dysregulated periostin expression is associated with various cardiomyopathies and typically increases in response to myocardial injury or stress (31). Furthermore, periostin supports angiogenesis, inhibits the apoptosis of vascular endothelial cells in scar tissue, improves endothelial function, and promotes fibroblast survival under hypoxic and nutrient-poor conditions, collectively facilitating tissue fibrosis and scar maturation (32–34).

Importantly, the dysregulation of periostin is not confined to nonneoplastic conditions. As noted earlier, its expression is frequently and markedly elevated in tumor tissues (10). Mechanistically, periostin increases the mechanical integrity of connective tissue by reinforcing interactions between ECM components (12). Through binding to integrins αvβ3 and αvβ5—which are expressed on osteoblasts, various normal cells, and malignant cells—periostin activates downstream signaling pathways, including focal adhesion kinase (FAK), phosphatidylinositol 3-kinase (PI3K), and protein kinase B (AKT/PKB), thereby promoting cell migration and invasion (5). These shared molecular mechanisms underscore the broader role of periostin in tissue remodeling and disease progression, providing a conceptual bridge to its functions within the tumor microenvironment.

## 4 Role of periostin in tumors

Accumulating evidence highlights the critical involvement of periostin in multiple oncogenic processes, including tumor cell proliferation, progression, migration, and epithelial-mesenchymal transition (EMT), as summarized in **Figure 2** (35). Mechanistically, periostin promotes local tumor invasion by inducing EMT and disrupting ECM homeostasis through fibrogenic activity, thereby fostering a prometastatic microenvironment that is associated with disease progression (36). Cancer-associated fibroblasts (CAFs) serve as the primary cellular source of periostin in tumors, and various periostin isoforms are differentially expressed across a spectrum of malignancies, including pancreatic, colorectal, breast, lung, and kidney cancers (5). Clinically, elevated periostin expression has been proposed as an independent candidate prognostic and predictive biomarker in several cancers (37).

**Figure 2:**
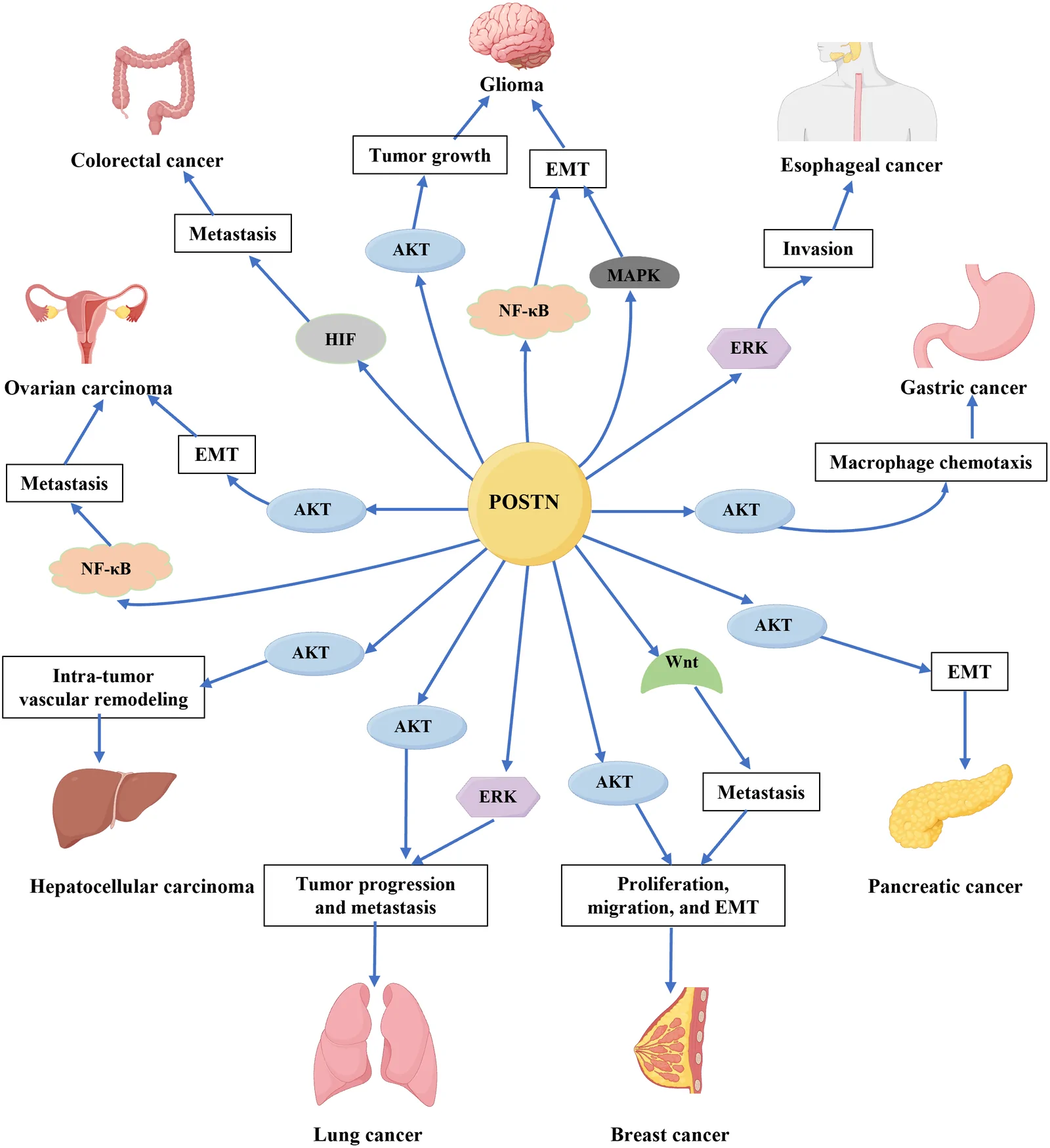
Schematic overview of periostin-mediated oncogenic signaling and functional outcomes in cancer. Periostin exerts its protumorigenic effects through interactions with multiple cell surface receptors, particularly integrins, leading to the activation of downstream signaling cascades, including the NF-κB, PI3K/AKT, MAPK/ERK, and ILK/AKT pathways. These signaling events collectively drive key oncogenic processes such as tumor cell proliferation, epithelial-mesenchymal transition (EMT), angiogenesis, and maintenance of cancer stemness. The resulting functional outcomes include increased tumor invasion and metastasis, therapeutic resistance, and immune evasion.

### 4.1 Periostin in gastrointestinal neoplasms

#### 4.1.1 Periostin in esophageal cancer

Elevated periostin expression is closely associated with poor prognosis and shorter overall survival in patients with esophageal squamous cell carcinoma (ESCC) (38). Bioinformatic analyses further confirmed that higher periostin levels are correlated with adverse outcomes and more advanced pathological stages in patients with esophageal cancer (EC) (39). In the experimental diagnostic setting, anti-periostin autoantibodies show promise as serological biomarkers for ESCC, with an area under the curve (AUC) of 0.638, a specificity of 90.27%, and a sensitivity of 27.57% (40). Notably, anti-periostin autoantibodies can distinguish patients with ESCC from normal controls across most clinical subgroups (40).

Functionally, a mechanistic study by Ishibashi et al. demonstrated that periostin promotes ESCC progression by increasing extracellular signal-regulated kinases (ERK)1/2 phosphorylation and upregulating the expression and activity of integrins as well as disintegrin and metalloproteinase 17 (ADAM17), a metalloproteinase critical for tumor initiation and invasion (41). In addition to being a canonical protein, the circular RNA circ-POSTN is also overexpressed in EC tissues and cell lines (42). Further mechanistic studies and mouse experiments revealed that the knockdown of circ-POSTN expression suppressed EC cell proliferation and invasion via the miR876-5p/FYN (FYN proto-oncogene, Src family tyrosine kinase) axis, while simultaneously promoting apoptosis and increasing radiosensitivity (42).

Furthermore, bioinformatic analyses conducted by Wang et al. indicated that periostin expression in EC is significantly correlated with marker genes of diverse tumor-infiltrating immune cells, including B cells, monocytes, tumor-associated macrophages (TAMs, both M1 and M2 macrophages), neutrophils, helper T cells, and regulatory T cells (Tregs) (43). These observations underscore the key role of periostin in shaping an immunosuppressive tumor microenvironment in esophageal cancer (43).

#### 4.1.2 Periostin in gastric cancer

Elevated periostin expression in gastric cancer (GC)-associated fibroblasts is linked to tumor metastasis and disease progression (44). Single-cell RNA sequencing analysis further revealed that extracellular matrix-enriched CAFs (eCAFs) expressing periostin are associated with increased M2 macrophage infiltration and poor patient survival (44), a finding corroborated by independent survival data from the Human Protein Atlas (**Figure 3a**).

**Figure 3:**
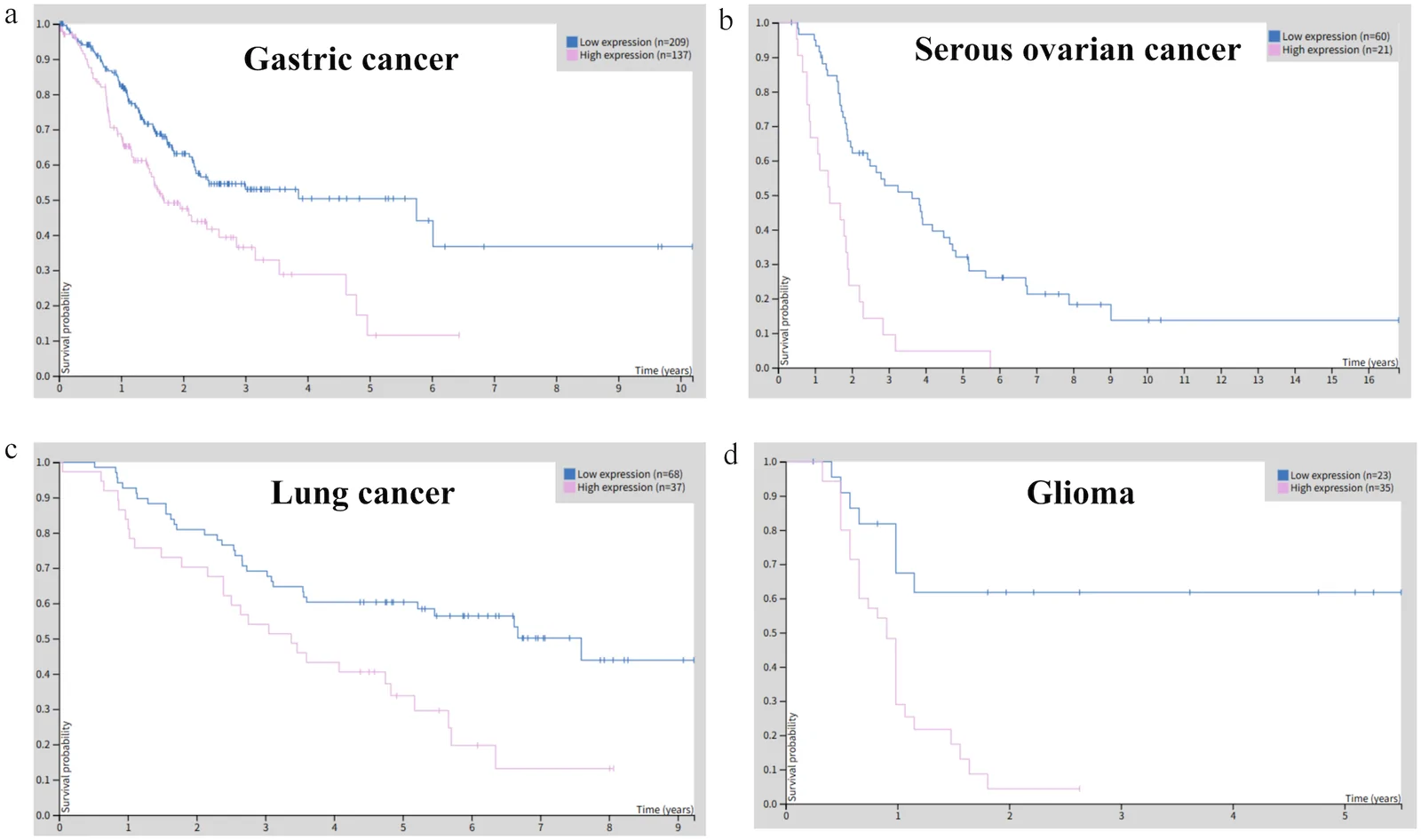
Prognostic significance of periostin expression across multiple cancer types. Kaplan-Meier survival curves demonstrate that high periostin expression is correlated with poor prognosis in patients with **(a)** gastric cancer (n = 209 with high expression vs. n = 137 with low expression), **(b)** serous ovarian cancer (n = 60 with high expression vs. n = 21 with low expression), **(c)** lung cancer (n = 68 with high expression vs. n = 37 with low expression), and **(d)** glioma (n = 23 with high expression vs. n = 35 with low expression). In all four tumor types, patients with low periostin expression had significantly better overall survival than those with high periostin expression did. Data were obtained from the publicly available Human Protein Atlas (HPA, https://www.proteinatlas.org/), and the survival curves presented herein were generated using the HPA survival analysis interface (https://www.proteinatlas.org/ENSG00000133110-POSTN/cancer). These data are derived from published, openly accessible datasets and do not represent unpublished original data generated by the authors. All survival analyses were performed using the online tool provided by the HPA database. The statistical significance of survival differences between the two groups was calculated using the log-rank test. High- and low-expression groups were defined using the best expression cut-off determined by the HPA survival analysis tool, which refers to the pTPM value that yields the maximal difference in survival between the two groups at the lowest log-rank P-value. The specific parameters for each cancer type are as follows: gastric cancer (cutoff: 107.14pTPM; median expression: 77.39pTPM; P-score: 0.00046), serous ovarian cancer (cutoff: 88.72pTPM; median expression: 40.48pTPM; P-score: 0.0000041), lung cancer (cutoff: 391.59pTPM; median expression: 297.62pTPM; P-score: 0.0022), and glioblastoma (cutoff: 14.08pTPM; median expression: 57.52pTPM; P-score:0.00012). All parameters are automatically generated by the HPA survival analysis interface, and no manual optimization of cutoffs was applied. These findings underscore the potential of periostin as a prognostic biomarker across diverse malignancies.

A study by You et al. utilizing both the TCGA-STAD dataset and real-world cohorts revealed that the abundance of POSTN^+^FAP^+^ (fibroblast activation protein) eCAFs may serve as a predictive biomarker for the response to immune checkpoint blockade (ICB) in GC (45). Mechanistically, these eCAFs secrete periostin to promote macrophage chemotaxis, thereby fostering an immunosuppressive microenvironment and driving ICB resistance (45). Given their pivotal role, POSTN^+^FAP^+^ eCAFs represent a promising therapeutic target for combination strategies aimed at improving ICB efficacy in GC (45).

#### 4.1.3 Periostin in colorectal cancer

Periostin is consistently overexpressed in colorectal cancer (CRC), with significantly higher levels in distant metastatic lesions than in corresponding primary tumors (46). *In vitro* studies have confirmed that periostin increases invasive behavior and can confer metastatic potential to otherwise nonmetastatic HEK 293T cells (46).

A bioinformatic study conducted by Gan et al. reported that elevated periostin expression in CAFs is correlated with advanced clinical stage and poor patient prognosis (47). Notably, dual positivity for CD70 and periostin in CRC-associated fibroblasts has been proposed as an indicator of more aggressive disease progression (48). Mechanistically, CD70 and periostin cooperate to induce an activated fibroblast phenotype, which increases the migratory and invasive capacity of CRC cells in coculture models, suggesting that the codetection of these markers may hold prognostic value (48).

The immunomodulatory function of periostin-expressing CAFs is being increasingly recognized. These POSTN^+^ CAFs are closely linked to intratumoral hypoxia and influence the response to immunotherapy in CRC (49). Bioinformatic analyses further revealed that periostin and SPOCK1 (SPARC/osteonectin, CWCV and Kazal-like domain proteoglycans 1) are coenriched in immune-related pathways involved in the regulation of tumor-infiltrating immune cells and immune checkpoint expression (47). Clinically, high levels of both proteins correlate positively with M2 macrophage polarization, neutrophil infiltration, and upregulation of immune checkpoint molecules, supporting their role in establishing an immunosuppressive microenvironment (47).

Mechanistically, periostin promotes programmed cell death protein 1 (PD-1) expression on TAMs via integrin-ILK (integrin-linked kinase)-NF-κB (nuclear factor kappa-light-chain-enhancer of activated B cells) signaling (50). Periostin deficiency markedly reduces PD-1^+^ TAM infiltration and, interestingly, increases the phagocytic capacity of TAMs (50). Importantly, the combined blockade of periostin and PD-1 expression significantly inhibited colorectal cancer growth by reducing the number of PD-1^+^ TAMs and increasing CD8^+^ T-cell infiltration (50). These findings suggest that dual targeting of periostin and immune checkpoints represents a promising therapeutic strategy, particularly for patients with refractory CRC who respond poorly to conventional chemotherapy or PD-1 monotherapy (50).

#### 4.1.4 Periostin in pancreatic cancer

Compared with normal pancreatic tissue, relevant bioinformatic studies have shown that periostin expression is significantly upregulated in pancreatic cancer tissue and is correlated with unfavorable prognosis and altered immune cell infiltration (51). Clinical analyses have confirmed that high periostin levels are strongly associated with poor outcomes in pancreatic ductal adenocarcinoma (PDAC) (52).

CAFs constitute a major stromal component of the pancreatic tumor microenvironment (TME) and encompass functionally heterogeneous subpopulations (53). Activated CAFs support tumor growth, angiogenesis, invasion, and metastasis through diverse signaling pathways (54). Notably, RNA sequencing and related experimental studies conducted by Neuzillet et al. reported that a combined transcriptional signature of periostin-positive CAFs and podoplanin (PDPN)-positive CAFs defines specific immune infiltration patterns within the TME (55). In tumors enriched with PDPN^+^ CAFs, the presence of POSTN^+^ CAFs shifts the immune landscape by suppressing T-cell activity while promoting monocyte and immunosuppressive M2-like macrophage activity (55).

Given that specific CAF subtypes have been proposed as predictors of immunotherapy response (56), the POSTN/PDPN RNA signature could help stratify patients into potential responders (high PDPN/low POSTN) and nonresponders (high POSTN), thus informing personalized treatment decisions (55). To further support this notion, Chen et al. demonstrated that periostin-expressing CAFs play a key role in remodeling the immunosuppressive pancreatic TME (51). Patients with low periostin expression exhibit better responses to cytotoxic T-lymphocyte-associated protein 4 (CTLA-4), PD-1, and programmed death ligand 1 (PD-L1) checkpoint inhibitors (51). In contrast, high periostin expression promotes an immunosuppressive milieu characterized by reduced T-cell infiltration, which likely undermines the efficacy of ICB in pancreatic cancer (51).

#### 4.1.5 Periostin in hepatocellular cancer

Bioinformatic analyses have demonstrated that periostin expression is significantly upregulated in hepatocellular cancer (HCC) tissues and cell lines, and elevated periostin levels correlate with shorter overall survival in patients (57).

By integrating spatial transcriptomics with scRNA-seq and multiplex immunofluorescence, Wang et al. identified that POSTN^+^ CAFs are markedly enriched and play a central role in modulating the immune landscape within the HCC tumor microenvironment (58). These CAFs suppress antitumor immunity by inhibiting CD8^+^ T-cell infiltration, which in turn impairs the response to immunotherapy (58). The abundance of POSTN^+^ CAFs may thus serve as a predictive biomarker for immunotherapy efficacy in patients with HCC (58). Notably, a distinct subset of hypoxic-metabolizing myofibroblasts (hmmyCAFs) has been identified as a major source of secreted periostin. These hmmyCAFs further impair antitumor immunity by suppressing immune checkpoint signaling and driving hypoxia-mediated glycolytic reprogramming of T cells (59). Clinically, both hmmyCAF activity and periostin expression correlate with reduced progression-free survival and increased resistance to immunotherapy in HCC patients (59).

In addition to direct immune modulation, POSTN^+^ CAFs promote tumor progression through paracrine signaling. They release migratory vesicles that deliver vascular endothelial growth factor A (VEGFA) and activate the VEGFR2-PI3K-AKT-eNOS (endothelial nitric oxide synthase) pathway, thereby increasing endothelial angiogenic activity and driving intratumoral vascular remodeling (60). Through these coordinated mechanisms—angiogenesis, immune evasion, and matrix remodeling—POSTN^+^ CAF-derived vesicles have emerged as key mediators of HCC aggressiveness and are closely linked to poor clinical outcomes and therapy resistance (60).

### 4.2 Periostin in ovarian cancer

Elevated periostin levels, which are frequently detected in the ascitic fluid of patients with ovarian cancer, together with increased macrophage infiltration, are significantly correlated with reduced recurrence-free survival, underscoring its clinical relevance (61). This prognostic association is further supported by independent survival data from the Human Protein Atlas, as shown in **Figure 3b**.

At the cellular level, exposure to elevated periostin increases stem-like properties and lipid synthesis in ovarian cancer cells, indicating its role in promoting tumor cell plasticity and metabolic adaptation (62). Lin et al. demonstrated that periostin facilitates immune invasion and stromal activation by polarizing macrophages toward an immunosuppressive M2 phenotype and activating CAFs through the integrin-dependent NF-κB and transforming growth factor-β2 (TGF-β2) pathways identified via *in vitro* experiments (63).

Notably, the prognostic significance of periostin depends on its cellular location. Systematic immunohistochemical evaluation revealed that high stromal periostin expression is strongly associated with adverse clinicopathological features and poor prognosis (64). In contrast, periostin expression within tumor cells appears to correlate with more favorable outcomes (64). This compartment-specific pattern highlights the complex and context-dependent role of periostin in ovarian cancer progression.

### 4.3 Periostin in thoracic neoplasms

#### 4.3.1 Periostin in lung cancer

Under physiological conditions, periostin expression in the lung is minimal, is primarily localized to the bronchial basement membrane, and is absent in alveolar macrophages (1). Its expression is markedly upregulated in lung cancer. Bioinformatic studies conducted by Sun et al. revealed that elevated periostin levels in lung adenocarcinoma (LUAD) correlate significantly with poor patient prognosis (**Figure 3c**), underscoring its value as a prognostic indicator (35). This protumorigenic role extends to non-small cell lung cancer (NSCLC), where cytoplasmic periostin expression is significantly higher in tumor tissues than in adjacent nonmalignant tissue (65). Clinically, periostin expression increases proportionally with tumor size, histological grade, and lymph node involvement, underscoring its association with disease progression (66).

*In vitro* studies confirmed that recombinant periostin increases LUAD cell invasion, migration, and proliferation while inhibiting apoptosis and promoting an EMT phenotype (35). Conversely, silencing periostin suppresses the migration and invasive capacity of lung cancer cells, an effect associated with the downregulation of matrix metalloproteinase-2 (MMP-2) and integrin-related signaling proteins (65). Mechanistically, periostin is associated with tumor advancement through multiple pathways. It interacts with VEGF-A and binds to integrins (αvβ3, αvβ5, and α6β4) on endothelial and cancer cells, activating key intracellular cascades such as those involving PI3K, AKT/PKB, FAK, and ERK. These signaling events collectively promote tumor cell survival, angiogenesis, invasion, and metastasis (66). Additionally, periostin contributes to NSCLC progression by upregulating TNFα-induced protein 6 (TNFAIP6) (67).

Through sequencing and immunohistochemical analyses, Chen et al. demonstrated that periostin-expressing myofibroblastic CAFs (myCAFs) are notably enriched in advanced NSCLC. These myCAFs exhibit transcriptomic signatures linked to increased invasion and ECM remodeling within the tumor stroma (68). Consequently, both periostin expression and an abundance of POSTN^+^ CAFs are significantly associated with advanced tumor stage and unfavorable prognosis in patients with NSCLC (68).

#### 4.3.2 Periostin in breast cancer

Immunohistochemical and cellular studies conducted by Ratajczak-Wielgomas et al. revealed that periostin expression is detected in both CAFs and tumor cells in patients with breast cancer (BC) (69). Previous studies have shown that periostin expression in breast cancer cells serves as an important prognostic indicator, associated with tumor size, lymph node status, and human epidermal growth factor receptor 2 (HER2) status (70). Notably, a fibroblast-derived short periostin isoform containing exon 17 but lacking exon 12 has been identified as a specific promoter of tumor growth and metastatic dissemination (71). Functionally, periostin contributes to BC metastasis by sustaining cancer stem cells through Wnt pathway activation (72).

Using enzyme-linked immunosorbent assay (ELISA) or electrochemiluminescence immunoassay, Jia et al. analyzed serum periostin levels in 242 participants, and revealed that elevated levels of soluble periostin show promise for early diagnosis, distant metastasis prediction and prognosis assessment, with performance comparable to that of the established biomarker CA153 (73). Jia et al. demonstrated that periostin exhibited the highest area under the curve (AUC) in differentiating healthy controls from patients with localized breast cancer (73). Furthermore, the combination of CA153 and carcinoembryonic antigen (CEA) with periostin significantly increased their respective AUC values (73). In addition, the efficacy of periostin in predicting distant metastasis in patients with breast cancer was comparable to that of CA153 (73).

In triple-negative BC (TNBC), periostin and periostin-expressing CAFs have emerged as particularly promising therapeutic targets. Proteomic and single-cell transcriptomic analyses revealed that periostin is markedly enriched in the “immune-excluded, stroma-high” subtype of TNBC, with single-cell RNA sequencing confirming its predominant expression in CAFs (74). Elevated periostin levels correlate with reduced tumor-infiltrating lymphocytes, underscoring its role in establishing an immune-excluded microenvironment (74). Mechanistically, CAF-derived periostin directly promotes TNBC cell proliferation (75). Additionally, TNBC-derived metastasis-associated lung adenocarcinoma transcript 1 (MALAT1) upregulates periostin expression to activate the Hippo/YAP (yes-associated protein) pathway, driving M2 macrophage polarization and facilitating tumorigenesis and metastasis *in vivo* (76). Given its central role in both tumor progression and immune evasion, targeting periostin-positive CAFs represents a promising therapeutic strategy to overcome immunosuppression and improve immunotherapy outcomes in patients with TNBC (74).

### 4.4 Periostin in glioma

Experimental results from Huizer et al. showed that periostin expression is significantly upregulated in specific glioma subtypes, notably pilocytic astrocytoma and glioblastoma (GBM), but remains low in grade II/III astrocytoma and oligodendroglioma (77). Furthermore, pericytes have been identified as the primary cellular source of periostin in human gliomas, where it contributes substantially to tumor-associated angiogenesis and vascular branching (77).

Mechanistically, periostin promotes glioma progression through multiple signaling pathways. Through bioinformatic analyses and *in vivo* and *in vitro* experiments, Wang et al. demonstrated that pentraxin 3 (PTX3) upregulates periostin expression and activates the MAPK (mitogen-activated protein kinase)/ERK (extracellular signal-regulated kinase) cascade, forming a PTX3-POSTN-MAPK axis that increases EMT and sustains drug-resistant glioma stem cells (GSCs), thereby driving invasion, metastasis, and recurrence (78). These findings collectively suggest that periostin is a promising prognostic indicator and therapeutic target in glioma (**Figure 3d**). In addition to its direct effects on tumor cells, periostin also modulates the immunosuppressive tumor microenvironment. Zhou et al. revealed that glioma stem cell-secreted periostin recruits M2-polarized tumor-associated macrophages from the peripheral blood, generating an immunosuppressive niche that accelerates tumor progression (79).

Isoform switching—defined as alterations in the expression of specific transcript isoforms—is frequently driven by cis-acting mutations in splice sites or trans-acting changes in splicing factors and is commonly associated with increased malignancy (80, 81). In cancer, this process can generate unique protein junction sequences known as tumor-enriched isoform junction antigens (TIAs) (81). Multiomics analysis of 587 patients with glioma revealed POSTN-203 as a GBM-enriched TIA candidate (82). This isoform exhibits HLA-A11-restricted immunogenicity and can elicit antitumor T-cell responses targeting TIA peptides (82). Consequently, vaccines targeting POSTN-203 and related TIAs may reduce the glioma stem cell burden and attenuate tumor self-renewal, vascularization, and therapeutic resistance (82, 83). These insights highlight POSTN-203 as a promising candidate for antigen-specific immunotherapy in glioma.

### 4.5 Summary

Collectively, the evidence presented above underscores periostin as a critical stromal-derived oncogenic regulator across a spectrum of malignancies. Its expression is consistently elevated in diverse cancers—including esophageal, gastric, colorectal, pancreatic, hepatocellular, ovarian, lung and breast carcinomas, and gliomas—and is strongly correlated with aggressive clinicopathological features and unfavorable patient prognosis. Beyond these well-characterized tumors, emerging evidence has implicated periostin in the pathogenesis of several other malignancies, as summarized in **Table 1**.

**Table 1:** The role of periostin in other tumors.

Cancer type	Study type	Correlated signaling pathway	POSTN function	Ref.
Cervical cancer	Bioinformatic analyses with experimental validation	–	Promotes proliferation, survival, and radioresistance	(84)
Kidney cancer	Experimental study	ILK/AKT/mTOR	Promotes EMT, angiogenesis	(85)
Thyroid cancer	Experimental study	Integrin-FAK-STAT3	Promotes proliferation	(86)
Craniopharyngioma	Experimental study	AKT	Activates EMT	(87)
Sarcoma	Bioinformatic analyses with experimental validation	–	Promotes monocytes differentiation into tumor-associated macrophages	(88)
Prostate cancer	Experimental study	TGF-β1/periostin/integrin αvβ3 axis	Promotes proliferation, invasion and bone metastasis	(89)
Bile duct cancer	Bioinformatic analyses	–	Promotes EMT	(90)

ILK, integrin-linked kinase; AKT, protein kinase B; mTOR, mechanistic target of rapamycin; EMT, epithelial–mesenchymal transition; FAK, focal adhesion kinase; STAT3, signal transducer and activator of transcription 3; TGF-β, transforming growth factor-β.

In addition to serving as a candidate prognostic biomarker, periostin actively promotes tumor progression through multiple interconnected mechanisms: it promotes EMT, increases angiogenesis, facilitates ECM remodeling, and sustains cancer stem-like properties (5). Periostin has also been reported to exert inhibitory effects on EMT in certain cancer types. For instance, Kim et al. demonstrated that POSTN upregulates E-cadherin expression, thereby suppressing the invasive and metastatic potential of bladder cancer cells (91). Particularly notable is its pivotal role within the tumor microenvironment. Periostin-expressing CAFs orchestrate a profoundly immunosuppressive niche by recruiting and polarizing M2-like macrophages, inhibiting cytotoxic CD8^+^ T-cell infiltration, and upregulating immune checkpoint molecules such as PD-1 (35). These activities collectively contribute to resistance against conventional therapies—including chemotherapy and radiotherapy—as well as to the limited efficacy of current immunotherapeutic approaches.

Importantly, these insights have spurred the exploration of novel therapeutic strategies. Targeting specific periostin isoforms, disrupting periostin-integrin signaling axes, or combining periostin inhibition with ICB has shown promising antitumor effects in preclinical models (35). The emerging role of periostin in modulating the responses to cancer immunotherapy is further detailed in **Table 2**. Future efforts to translate these strategies into clinical practice may provide new avenues for overcoming therapy resistance and improving outcomes in patients with periostin-high tumors.

**Table 2:** The role of periostin in tumor immunotherapy.

Cancer type	Study type	Immunotherapy type	Periostin function	Ref.
Glioma	Bioinformatic analyses with experimental validation	Cancer vaccines	POSTN-203 serves as a tumor-enriched isoform junction antigen for immunotargeting	(82)
Esophageal cancer	Bioinformatic analyses	–	Expression correlates with marker genes of various tumor-infiltrating immune cells	(43)
Gastric cancer	Experimental study	ICB	Contributes to an immunosuppressive microenvironment and is associated with ICB resistance	(45)
Colorectal cancer	Bioinformatic analyses with experimental validation	ICB	Fosters an immunosuppressive microenvironment	(47)
Pancreatic cancer	Bioinformatic analyses	ICB	Supports an immunosuppressive tumor microenvironment characterized by reduced T-cell infiltration	(51)
Liver cancer	Bioinformatic analyses with experimental validation	ICB	Suppresses immune checkpoint signaling	(59)
Breast cancer	Experimental study	–	Facilitates immune evasion	(74)

POSTN, periostin; ICB, immune checkpoint blockade.

## 5 Signaling pathways mediated by periostin

Periostin exerts its diverse biological effects largely through the modulation of key intercellular signaling pathways. This section summarizes the current evidence on the crosstalk between periostin and two major signaling axes—NF-κB and AKT—in both inflammatory diseases and cancers.

### 5.1 The periostin/NF-κB axis

NF-κB is a ubiquitous transcription factor that plays a pivotal role in regulating inflammation, immune responses, and tumorigenesis (92, 93). Periostin has been established as an upstream activator of the NF-κB pathway across various cell types, leading to the production of proinflammatory cytokines and chemokines (94).

Under nonneoplastic conditions, periostin promotes intervertebral disc degeneration by driving inflammation and apoptosis of nucleus pulposus cells via NF-κB activation (95, 96). Similarly, it contributes to osteoarthritis progression through NF-κB-dependent mechanisms (97). In oncology, activation of the NF-κB pathway by periostin is closely linked to tumor malignancy and therapy resistance (63). For instance, in CRC, periostin upregulates PD-1 expression on TAMs via the integrin-ILK-NF-κB cascade, thereby shaping an immunosuppressive microenvironment (50). Similarly, in HCC, periostin promotes metastasis by upregulating C-C motif chemokine ligand 2 (CCL2) in endothelial progenitor cells through the αvβ3/ILK/NF-κB pathway (98). This axis also operates in ovarian cancer, where periostin-mediated NF-κB activation facilitates the recruitment and polarization of M2-like macrophages and CAFs (63). Furthermore, in GBM, periostin-induced NF-κB signaling increases EMT and chemoresistance (99).

Overall, the periostin/NF-κB axis serves as a critical regulator of progression, metastasis, and therapeutic resistance in both inflammatory diseases and multiple cancers.

### 5.2 The periostin/AKT axis

The PI3K/AKT pathway is a central regulator of cell survival, proliferation, metabolism, and motility, and is frequently activated in human diseases (100, 101). Periostin interacts with several integrin receptors (e.g., αvβ1, αvβ3, or αvβ5) to activate this pathway, thereby mediating diverse pathological processes (94, 102).

#### 5.2.1 Periostin and the PI3K/AKT pathway

Following myocardial infarction, periostin activates PI3K-AKT signaling via integrins αvβ1, αvβ3, or αvβ5 on cardiomyocytes and endothelial cells, promoting tissue regeneration and angiogenesis (102). In cancer, periostin-mediated PI3K-AKT activation is associated with aggressive phenotypes across multiple tumor types. For example, in head and neck cancer, bone marrow mesenchymal stem cell-derived periostin activates the PI3K/AKT/mTOR (mechanistic target of rapamycin) pathway to increase tumor cell proliferation, invasion, and survival (103). In ESCC, recombinant periostin promotes cell survival and migration via AKT phosphorylation in an integrin β4-dependent manner (104). Similarly, in GC, podoplanin-positive CAFs secrete periostin, which induces PI3K/AKT phosphorylation in a positive feedback loop that sustains periostin production and facilitates metastasis (105). In addition, the PI3K/AKT pathway has been implicated in periostin-driven osteosarcoma proliferation and metastasis (106), CRC cell proliferation and migration (107), and tumor angiogenesis across various contexts (37).

#### 5.2.2 Periostin and the ILK/AKT pathway

ILK functions as a critical downstream mediator of integrin signaling and is frequently overexpressed in various cancers, where it promotes tumor progression (57). Periostin can signal through ILK to activate AKT and its downstream effectors. For instance, periostin increases the migration and invasion of human endometrial stromal cells via the ILK1/AKT pathway (108). In HCC, trimethylamine N-oxide induces periostin expression, which subsequently activates the ILK/AKT/mTOR axis to promote inflammation and tumorigenesis (57). Similarly, high periostin expression activates the ILK/AKT/mTOR pathway to drive EMT and angiogenesis, whereas POSTN knockdown reverses these effects by modulating the expression of EMT markers in kidney cancer (85). These findings underscore that the ILK/AKT pathway is a key conduit for periostin-mediated oncogenic signaling.

## 6 Periostin and resistance to immune checkpoint blockade

ICB, particularly therapies targeting the PD-1/PD-L1 axis, has revolutionized the treatment of multiple advanced solid tumors (109). However, therapeutic efficacy remains heterogeneous and is largely dictated by the state of the TME (110). A favorable response to ICB critically depends on the presence and activity of CD8^+^ effector T cells within the tumor, which these agents aim to reactivate (111). The immunosuppressive nature of the TME often limits CD8^+^ T-cell infiltration and function, thereby undermining the efficacy of ICB (110).

### 6.1 Periostin-mediated immune evasion and ICB resistance

Emerging evidence implicates periostin as a key modulator of ICB resistance through its effects on the immune landscape. As illustrated in **Figure 4**, periostin indirectly restricts effector T-cell activity by fostering a microenvironment that promotes the infiltration of immunosuppressive macrophages while limiting CD8^+^ T-cell recruitment in colorectal cancer (50). In experimental models, periostin deficiency significantly reduces the infiltration of PD-1^+^ TAMs and concurrently increases CD8^+^ T-cell accumulation within the TME (50). By alleviating TAM-mediated immunosuppression, periostin inhibition synergizes with ICB to unleash a more effective antitumor immune response (50). Furthermore, bioinformatic research conducted by Chen et al. revealed that periostin interacts with integrins and growth factors to activate pathways such as TGF-β signaling, thereby promoting the differentiation of Tregs (70). The expansion of Tregs suppresses overall antitumor immunity and facilitates tumor immune escape (70).

**Figure 4:**
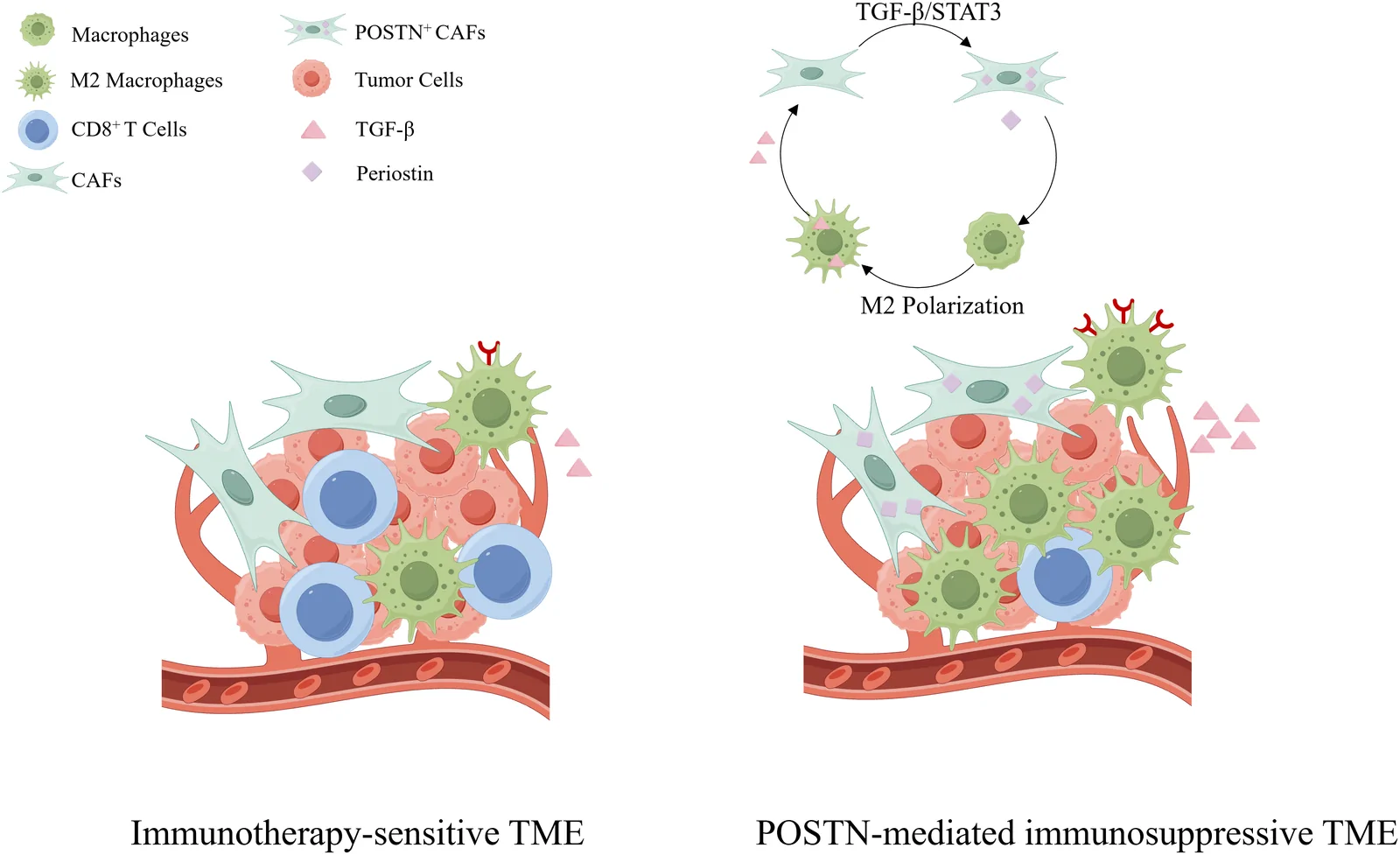
Mechanism of immunotherapy resistance mediated by periostin.

### 6.2 Role of POSTN^+^ CAFs in ICB resistance

CAFs are central architects of the immunosuppressive TME. Through the secretion of cytokines and extracellular vesicles, as well as the formation of a physical barrier, CAFs potently inhibit the infiltration and activity of cytotoxic T lymphocytes (112–114). Among the various CAF subsets, those expressing periostin (POSTN^+^ CAFs) have emerged as key mediators of stromal-tumor crosstalk and immune evasion in non-small cell lung cancer (115), with multifaceted roles illustrated in **Figure 5**. Accumulating evidence directly links POSTN^+^ CAFs to ICB resistance. In liver metastasis from colorectal cancer, Liu et al. identified that POSTN^+^ CAFs engage with TGF-β signaling to suppress T-cell function, by the analysis of clinical samples and databases (116). Experimental knockdown of periostin in fibroblasts reduces TGF-β expression and restores T-cell activity (109). In murine models, tumors rich in periostin-expressing fibroblasts exhibit resistance to PD-1 blockade, whereas POSTN depletion reverses this resistance, highlighting the role of the TGF-β-POSTN axis as a viable therapeutic target in CAF-dominant tumors (109). Wang et al. constructed a tumor microenvironment atlas of clear cell renal cell cancer and revealed that hypoxia and TGF-β/STAT3 (signal transducer and activator of transcription 3) signaling drive CAFs toward an ECM-remodeling, periostin-high phenotype (117). These fibroblasts secrete periostin to recruit and polarize CCL3^+^ macrophages, which then release TGF-β, platelet-derived growth factor B (PDGFB), and secrete phosphoprotein 1 (SPP1), creating an immune-excluded, suppressive microenvironment (117). Furthermore, clinical analyses of TCGA-KIRC and ICB-treated cohorts confirmed that this axis is correlated with ICB resistance and poor prognosis in clear cell renal cell carcinoma (117).

**Figure 5:**
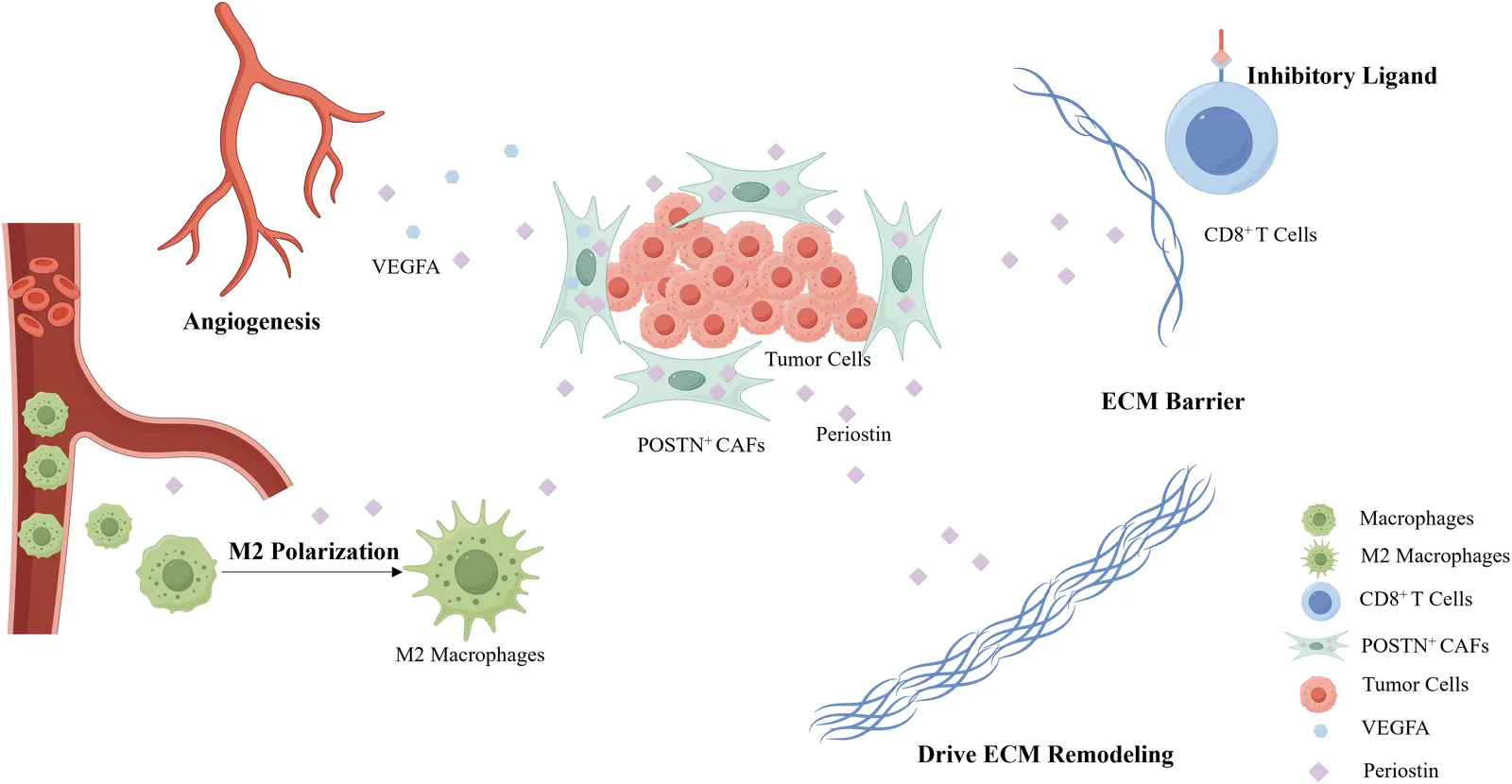
Multiple roles of POSTN^+^ CAFs in the tumor microenvironment.

### 6.3 Therapeutic strategies targeting periostin to increase ICB efficacy

Given its pivotal role in immune evasion, periostin has emerged as a promising target for combination strategies with ICB. Preclinical studies have demonstrated that blocking periostin function using various approaches increases antitumor immunity and improves responses to immunotherapy.

One notable strategy involves combining ICB with periostin pathway antagonists. Sun et al. demonstrated that concurrent administration of PD-L1 blockade and RGD—a periostin pathway antagonist—significantly increases therapeutic efficacy in lung adenocarcinoma (35). In a mouse subcutaneous tumorigenesis model, this combination therapy not only inhibited tumor growth but also increased CD8^+^ T-cell infiltration and favorably shifted the M1/M2 macrophage ratio within the TME (35). Several periostin-targeting biologics have also shown promise as monotherapies or potential combination agents. A neutralizing anti-periostin antibody (PN1-Ab) targeting the polypeptide encoded by exon 17 has been shown to inhibit primary tumor growth and metastatic lesions in BC mouse models, reduce bone degradation, and increase survival rates (118). In parallel, a benzyl-d(U)TP-modified DNA aptamer targeting human periostin similarly suppressed tumor growth and metastasis in BC models (70). Additional antibody-based approaches have focused on disrupting specific molecular interactions. The monoclonal antibody MPC5B4 was engineered to block the interaction between periostin and αvβ3 integrin by targeting amino acids 136~151 within the FAS1–1 domain of periostin, thereby inhibiting its protumorigenic functions (70). Furthermore, the anti-periostin monoclonal antibody MZ-1 has demonstrated efficacy in reducing the metastatic burden in ovarian cancer mouse models (70).

### 6.4 Summary

In summary, POSTN^+^ CAFs play a pivotal role in orchestrating a multifactorial immunosuppressive program within the TME. Through the modulation of macrophage polarization, maintenance of TGF-β signaling, and activation of integrin-dependent pro-survival pathways, these cells establish a barrier to T-cell infiltration and function. Targeting periostin—whether via neutralizing antibodies, aptamers, or pathway antagonists—represents a promising combinatorial strategy to overcome ICB resistance and enhance the efficacy of immunotherapy (70).

Despite the encouraging prospects of periostin-targeted research, no clinical trials specifically directed against periostin have been initiated to date. Moreover, substantial challenges persist, including target specificity, resistance driven by stromal pathway redundancy, and the lack of robust evidence for stratifying patient populations based on clinical response. Beyond its elevated expression in the tumor stroma, periostin also plays essential roles in normal physiological processes, such as tissue repair, wound healing, and bone fracture healing, as previously discussed. Consequently, systemic periostin-targeting therapies may inadvertently affect normal tissues. Furthermore, given the context-dependent functions of periostin across different tumor types, future clinical applications will likely require precise identification of responsive subpopulations—particularly those with “stroma-rich, immune-excluded” phenotypes—to maximize therapeutic benefits. This heterogeneity poses a major challenge to single-agent targeted strategies, underscoring the need for refined patient stratification in clinical translation.

## 7 Conclusions and future perspectives

In summary, periostin functions as a critical oncogenic regulator across multiple cancer types. It promotes malignant progression by stimulating tumor cell proliferation, inducing angiogenesis, sustaining cancer stemness, and promoting EMT. At the molecular level, periostin orchestrates EMT by upregulating vimentin and N-cadherin expression while downregulating E-cadherin expression, thereby increasing cellular migration and invasion (119, 120). Concurrently, it activates the AKT/PKB pathway to promote angiogenesis and metastasis while protecting tumor and endothelial cells from stress-induced apoptosis (121). Clinically, elevated periostin expression—in both the tumor stroma and epithelial compartments—is consistently correlated with aggressive tumor behavior, advanced disease stage, poor prognosis, and poor overall survival, underscoring its strong potential as a candidate prognostic biomarker (10). However, owing to the limited number of available studies and the inconclusive nature of current findings, definitive conclusions regarding the clinical utility of serum periostin as a prognostic biomarker cannot yet be established. Accordingly, large-scale prospective studies are warranted to further clarify its clinical relevance.

While earlier research predominantly focused on the tumor-promoting functions of periostin, recent advances in immunotherapy have shifted attention toward its role in modulating antitumor immunity and driving therapeutic resistance. These oncogenic mechanisms and clinical associations provide a foundation for understanding how periostin contributes to immune evasion. Nevertheless, the precise molecular circuits through which periostin orchestrates immune evasion remain incompletely elucidated.

Building on these insights, emerging therapeutic strategies aimed at targeting periostin have shown promise in preclinical models. As summarized in **Figure 6**, these approaches include neutralizing antibodies targeting specific periostin isoforms, disruption of periostin-integrin signaling axes, and combination regimens with ICB. Future investigations should prioritize delineating the underlying pathways—particularly the crosstalk between periostin-expressing CAFs, immune cells, and checkpoint molecules—and rigorously evaluate combinatorial strategies that concurrently target periostin signaling and established immune checkpoints. Such approaches hold substantial promise for overcoming resistance and improving clinical outcomes in patients with periostin-high malignancies.

**Figure 6:**
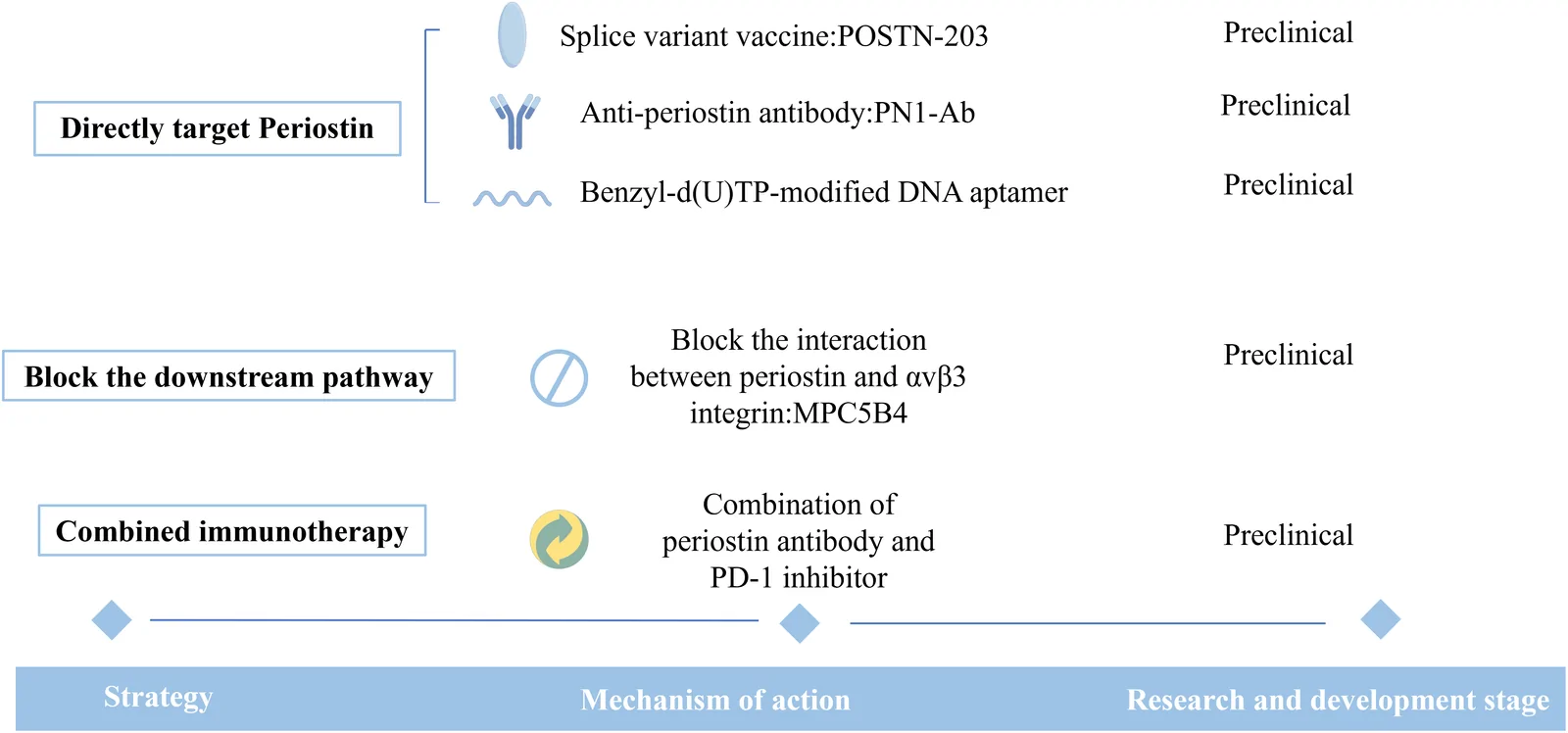
Schematic summary of targeted therapeutic strategies targeting periostin and their clinical prospects. This figure illustrates current approaches for targeting periostin in cancer therapy, including neutralizing antibodies (e.g., PN1-Ab), DNA aptamers, and small molecule antagonists that disrupt periostin-integrin interactions. These strategies can be employed as monotherapies or in combination with immune checkpoint blockade to overcome immunosuppression and increase antitumor immunity.
